# Combat sports and wellbeing: advancing health and inclusion in athletes and practitioners. An opinion paper

**DOI:** 10.3389/fpsyg.2025.1587672

**Published:** 2025-04-22

**Authors:** Simone Ciaccioni, Youngjun Lee, Flavia Guidotti, Nemanja Stankovic, Elena Pocecco, Pascal Izzicupo, Laura Capranica

**Affiliations:** ^1^Department of Education and Sport Sciences, Pegaso Telematic University, Naples, Italy; ^2^Department of Movement, Human and Health Sciences, University of Rome “Foro Italico,” Rome, Italy; ^3^Department of Kinesiology, Michigan State University, East Lansing, MI, United States; ^4^Department of Human Sciences and Promotion of Quality of Life, University of Rome “San Raffaele,” Roma, Italy; ^5^Faculty of Sport and Physical Education, University of Niš, Niš, Serbia; ^6^Department of Sport Science, University of Innsbruck, Innsbruck, Austria; ^7^Department of Medicine and Aging Sciences, University of Chieti-Pescara “Gabriele D'Annunzio”, Pescara, Italy

**Keywords:** martial arts, benefits, research, psycho-social impact, risks, skills, quality of life, community

## 1 Introduction

Historically, combat sports have been predominantly conceptualized within the framework of elite competition, emphasizing physical aptitude, technical proficiency, and strategic execution (James et al., 2016; Slimani et al., 2017; Chaabene et al., 2018). Despite their traditional and peculiar constitutions and developments, disciplines such as judo, karate, taekwondo, wrestling, fencing, boxing, and mixed martial arts have commonly been associated with high-performance athletes striving for competitive excellence on national and international stages (James et al., 2016; Franchini et al., 2011; Lystad et al., 2020; Jäggi et al., 2015). However, contemporary discourse increasingly recognizes their expansive role in contributing to physical and psychological wellbeing, and social inclusion of the practitioners (Valdes-Badilla et al., 2021; Zou et al., 2018). This paradigmatic shift underscores the capacity of combat sports to function as inclusive and accessible modalities for fostering multidimensional health benefits across diverse populations, including individuals with disabilities and other marginalized groups (Gutierrez-Santiago et al., 2023; Palumbo et al., 2023; Rajan, 2015; Morales et al., 2022; Pierantozzi et al., 2022).

The interdisciplinary exploration of physical activity and health highlights the intricate interrelationship between structured sports engagement and holistic wellbeing (Rato Barrio et al., 2021). Whilst conventional team and individual sports have long been acknowledged for their physiological and psychosocial benefits, combat sports exhibit distinct characteristics that might amplify these advantages (Malm et al., 2019; Garcia-Falgueras, 2015). Within combat sports, the synergistic interplay of rigorous physical conditioning, the cognitive engagement, adherence to rules, competition dynamics, respect, externalizing emotions regulation, are intertwined with pedagogical and philosophical values, thus presenting a unique framework for enhancing psychological resilience, cognitive adaptability, and emotional control. Consequently, the systematic practice of combat sports has been increasingly examined as a way of promoting mental health, stress modulation, and social cohesion (Ciaccioni et al., 2024a; Kujach et al., 2022).

The inclusive nature of many combat sports programmes further accentuates their relevance in dismantling stereotypes, facilitating integration, and fostering equity and social integration (Ciaccioni et al., 2024e; Jeong et al., 2021; Descamps et al., 2024). In fact, they demonstrated significant adaptability to accommodate individuals with disabilities (e.g., physical impairments, developmental, emotional, intellectual disorders), thereby ensuring equitable access and fostering empowerment (Gutierrez-Santiago et al., 2023; Salgado and Jauregui, 2022). Adapted judo, para-taekwondo, and other modified combat disciplines provide individuals with disabilities a structured platform to engage in physical activity, cultivate self-efficacy, and develop meaningful social connections within a supportive and adaptive environment (Lee et al., 2025). From a public health perspective, the integration of combat sports within community-based health initiatives offers a compelling opportunity to engage populations that may not traditionally participate in structured physical activity programmes (Valdes-Badilla et al., 2021; Zou et al., 2018; Ciaccioni et al., 2024e). The distinctive accessibility of combat sports, which cater to practitioners of all skill levels (e.g., from novices to elite athletes) sets them apart from many other sports. While their structured progression, adaptability, and emphasis on holistic development make them a viable option for lifelong and intergenerational participation (Ciaccioni et al., 2024e, 2019b, 2024b), the mentorship and pedagogical frameworks cultivate positive role modeling, discipline, and intrinsic motivation, which are integral for sustaining long-term adherence to health-promoting behaviors (Zou et al., 2018).

Furthermore, the intersection between combat sports and mental health has increasingly emerged as a focal point within academic and clinical research (Ciaccioni et al., 2024a) with empirical evidence suggesting an association between participation and enhanced self-regulation and self-efficacy, and reduction in anxiety and depressive symptomatology (Zou et al., 2018; Piskorska et al., 2016; Mojtahedi et al., 2023). In necessitating sustained focus, adaptability, and emotional equilibrium, combat sports inherently require cognitive and affective demands, which align closely with established psychological frameworks that underpin mental wellbeing (Healey et al., 2025; Sullivan et al., 2024). Moreover, the integration of mindfulness techniques, stress management strategies, and resilience-building paradigms within combat sports training substantiates their potential as a non-pharmacological intervention for addressing various mental health challenges (e.g., autism spectrum and oppositional defiant disorders; Pedrini and Jennings, 2021; Andreato et al., 2022).

Despite these advantages, the discourse surrounding combat sports and wellbeing necessitates a critical examination of inherent risks and potential challenges. Issues related to injury risk, hypercompetitive environments, eating disorders, sexual harassment, and the psychological stressors associated with high-intensity training warrant careful scrutiny (Hammami et al., 2018; Bromley et al., 2018; Gauthier, 2009; Barcelos et al., 2024; Mathisen et al., 2022). The implementation of evidence-based injury prevention protocols, the establishment of ethically responsible coaching methodologies, and the promotion of safe training environments are imperative to ensure that the benefits of combat sports are maximized while minimizing adverse outcomes.

Against this backdrop, this opinion paper seeks to examine the role of combat sports in advancing health and social inclusion among athletes and practitioners. Through a synthesis of contemporary empirical findings, theoretical paradigms, and applied insights, this paper aims to contribute to the evolving discourse on the potential of combat sports as a catalyst for holistic wellbeing. By delineating the multidimensional impact of combat sports on physical, psychological, and social health, this paper endeavors to underscore their transformative potential as an instrument for fostering individual and community wellbeing within different populations.

## 2 Discussion

**Figure 1:**
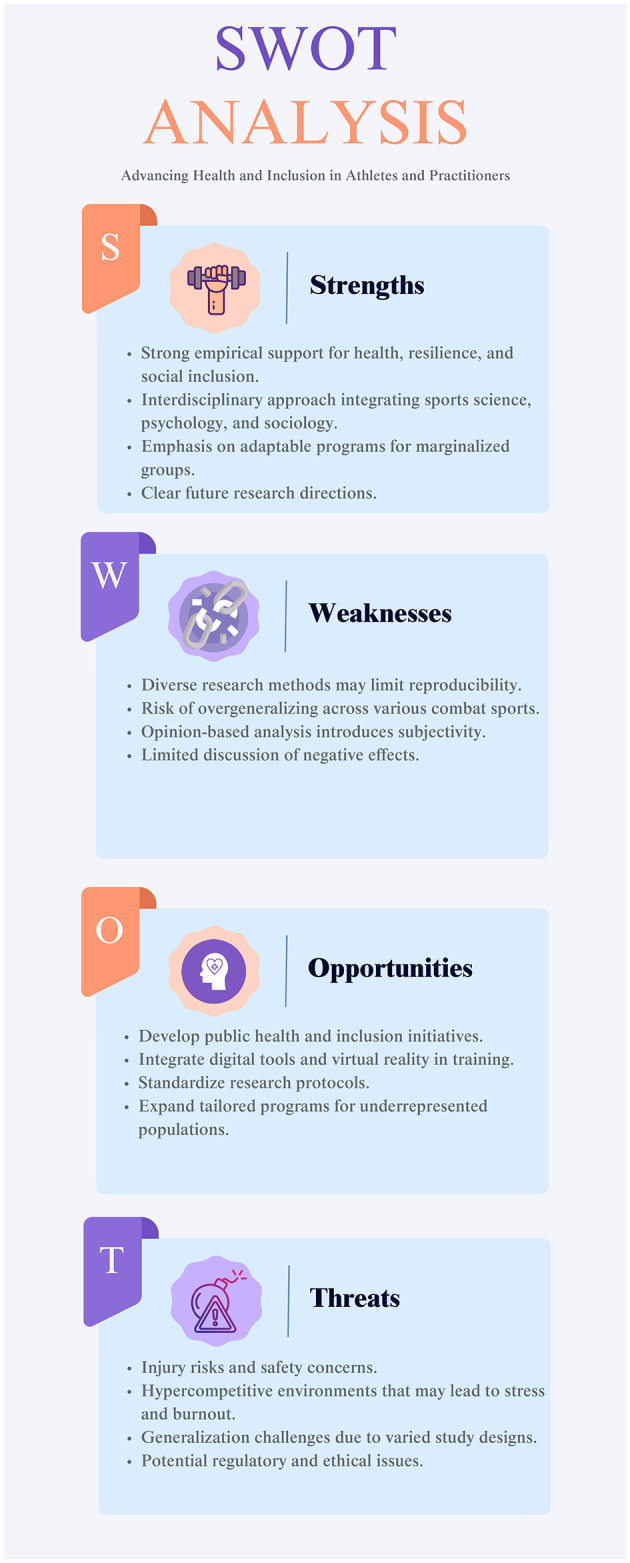
Strengths, weaknesses, opportunities, and threats (SWOT) analysis for combat sports.

Whilst an expanding body of research and an evolution in scholarly discourse is recognizing the combat sports' broader implications for holistic wellbeing (Pedrini and Jennings, 2021; Klimczak et al., 2015), a rigorous evaluation of the investigation methodologies, the validity of hypotheses, and the translational potential of recent findings is necessary to contextualize their significance within the sports and public health sciences, considering their strengths, weaknesses, opportunities, and threats (Figure 1).

Empirical evidence robustly shows the positive impact of combat sports on physical fitness, motor coordination, and cardiovascular health (Chaabene et al., 2018; Origua Rios et al., 2018). These benefits are attributed to the high-intensity, intermittent nature of combat sports training, which enhances aerobic and anaerobic endurance, muscular strength, and neuromuscular control (Eckstein et al., 2022). However, concerns regarding injury risk, particularly in striking and contact-intensive disciplines such as boxing, taekwondo, and mixed martial arts, necessitate continued research into injury mitigation strategies, particularly those targeting concussion and repetitive head trauma (Jäggi et al., 2015; Barcelos et al., 2024; Lota et al., 2022; Pocecco et al., 2013). Moreover, many combat sports have developed styles with reduced or simulated contact to minimize injury risk. For example, the French “boxe éducative” emphasizing technique and control, penalizing any violent behaviors (Cougoulic et al., 2003) and the value and application of kata (i.e., forms; prearranged, pattern practices) to learning and adopting judo technique in a safe way educating the athlete culturally, to enrich her/him as a person (Calmet et al., 2024).

Beyond physical health, recent studies highlight the psychological benefits of combat sports, including reductions in anxiety and depression and improvements in self-efficacy, emotional regulation, resilience, and stress management (Mojtahedi et al., 2023; Matsumoto et al., 2009; Stankovic et al., 2021; Slimani et al., 2018; Ciaccioni et al., 2024d). Therefore, combat sports-based interventions for individuals with mental health conditions have yielded promising outcomes (Lee et al., 2025). Despite these encouraging findings, variability in study designs, participant demographics, and intervention protocols limits their external validity, underscoring the need for further rigorously controlled investigations. Furthermore, some authors claimed that combat sport athletes might present symptoms of low energy availability and high anxiety levels associated with competition- and injury-related psychological stressors, deficits in executive functions and neuropsychological impairments associated with occurrence of concussions, disordered eating and eating disorders associated with weight-loss, and might suffer offensive, frightening, hostile, degrading, humiliating experiences, or sexual harassment, which urge safeguarding actions (Hammami et al., 2018; Bromley et al., 2018; Gauthier, 2009; Barcelos et al., 2024; Mathisen et al., 2022).

The role of combat sports in fostering social inclusion has gained empirical support, particularly in programmes aimed at individuals with disabilities and marginalized communities (Ciaccioni et al., 2024b; Boguszewski and Torzewska, 2011). For instance, a recent systematic review shows that judo interventions adapted for intellectual disabilities help improve social integration and self-perception and enhance participants' quality of life (Pečnikar Oblak et al., 2020). The development of para-combat sports demonstrates enhanced physical and motor abilities while providing psychosocial benefits to various populations with different disabilities, promoting social integration, self-perception, and community belonging (Connor et al., 2018; Kasum et al., 2011). However, longitudinal research is needed to assess the long-term retention rates and sustainability of these benefits. Furthermore, there is a need of studies focused on the most appropriate adapted rules to achieve a fairer competition for ensuring a sense of success in individuals with physical, emotional, mental, hearing or visual impairments participating in adapted sports competitions at local, national, and international levels.

Methodological approaches in combat sports research encompass experimental, longitudinal, qualitative, and systematic review designs. While randomized controlled trials remain the gold standard for establishing causality, their application in combat sports research is constrained by ethical concerns, logistical challenges, and the inherently dynamic nature of training environments (Kordi, 2009; Drid, 2017). Consequently, many studies rely on observational designs, which, despite their value in identifying associations, are susceptible to confounding variables and biases (Ciaccioni et al., 2019b; Grimes and Schulz, 2002).

Qualitative methodologies have provided critical insights into the lived experiences of combat sports practitioners, offering perspectives on psychological and social dimensions that are often overlooked in quantitative studies (Healey et al., 2025). Ethnographic research has been particularly instrumental in elucidating the role of combat sports in shaping identity, discipline, and personal development (García and Spencer, 2013). However, limitations in reproducibility and generalizability highlight the need for mixed-methods approaches to generate a more comprehensive understanding of combat sports' impact (Ciaccioni et al., 2024b; Smith and McGannon, 2017).

Additionally, the incorporation of biometric and neurocognitive assessments, such as heart rate variability analysis, functional MRI, and salivary cortisol measurements, has advanced our understanding of the physiological and psychological mechanisms underlying combat sports participation (Ciaccioni et al., 2024d; Morales et al., 2013; Sethi and Neidecker, 2023). Despite their objective precision, these techniques often face challenges related to cost, accessibility, and limited sample sizes, necessitating the development of scalable and cost-effective methodologies for broader research application.

Finally, recent studies show that virtual reality (VR) technology and digital platforms are increasingly becoming part of combat sports training methods. Due to COVID-19 restrictions, martial arts schools and organizations implemented hybrid or online training models, which allowed athletes to stay engaged. VR boxing programmes provide users with virtual sparring simulations to enhance their motor skills without needing physical interaction. Initial results indicate that VR training enhances response behavior in karate athletes (Witte et al., 2022). Digital adaptations offer great potential, especially for people with limited mobility or remote locations. However, further studies are necessary to prove their enduring effects on physical health and psychological and social aspects (Witte et al., 2022; Xu et al., 2021; Li et al., 2025).

## 3 Strengths and weaknesses of scientific hypotheses

The hypothesis that combat sports confer multidimensional health benefits is strongly supported by empirical evidence spanning physiological, psychological, and social domains (Zou et al., 2018; Channon and Jennings, 2014; Ciaccioni et al., 2019a; Torres-Luque et al., 2016). The integration of physical exertion, cognitive engagement, and structured discipline inherent in combat sports aligns with established theories of exercise psychology, neuroplasticity, and social identity formation (Krabben et al., 2019; Anastasiou et al., 2024). This multidimensional perspective provides a robust theoretical foundation for advocating combat sports as a health-promoting activity.

Nevertheless, several limitations warrant consideration. The heterogeneity of disciplines, which vary highly in intensity, contact level, and training methodologies, is often inadequately addressed in research, leading to overgeneralized conclusions (Ciaccioni et al., 2024a; Eckstein et al., 2022). Often underexamined, individual differences in personality, motivation, and previous trauma history may strongly moderate the psychological outcomes of combat sports participation (Barcelos et al., 2024; Bojanic et al., 2019; Ziv and Lidor, 2013).

Additionally, a research focus is needed on concerns regarding the potential for adverse psychological effects (e.g., anxiety, depression, disordered eating behaviors, burnout, and decreased self-esteem), particularly in competitive environments where performance pressure, extreme weight-cutting practices, and aggressive coaching styles are prevalent (Barley et al., 2019; Lafuente et al., 2021). Indeed, a balanced perspective that considers both benefits and risks is essential for the development of evidence-based recommendations (Palumbo et al., 2023; Qi et al., 2024).

## 4 Future directions

To enhance the field, future research should prioritize well-structured longitudinal studies that assess the long-term impact of combat sports participation on physical, psychological, and social health. Standardization of outcome measures, intervention protocols, and participant demographics would facilitate cross-study comparisons and strengthen the reliability of findings (Piggott et al., 2019; Halperin et al., 2018). Moreover, interdisciplinary collaborations incorporating sports science, psychology, and sociology could provide a more holistic perspective on combat sports' broader implications on practitioners (Elferink-Gemser et al., 2018).

From a policy perspective, to yield valuable insights into combat sports' practical applications research should investigate their efficacy within public health, educational, and rehabilitation initiatives, particularly for underserved and vulnerable populations (Lee et al., 2025; Dortants et al., 2016; Ciaccioni et al., 2024c).

Furthermore, while safety remains a primary concern, advancements in protective equipment, training methodologies, education for athletes, coaches, referees and tournament directors, and regulatory frameworks should be continually evaluated to optimize benefits while mitigating risks (Lota et al., 2022; Pocecco et al., 2013; Sethi and Neidecker, 2023; Bakirtzis et al., 2024; Pocecco et al., 2024). Ethical considerations, particularly concerning athlete wellbeing and inclusive participation, should remain a central focus in both research and practical implementation (Ciaccioni et al., 2024a; Gauthier, 2009; Mathisen et al., 2022).

Therefore, key research challenges to be addressed are:

- Injury risk and safety: a need for injury prevention strategies, especially for concussions and head trauma in striking sports.
- Psychological wellbeing: risks of stress, anxiety, burnout, and negative self-perception in competitive environments.
- Inclusion and accessibility: a need for more research on long-term social and psychological benefits for marginalized groups and individuals with disabilities.
- Methodological limitations: lack of standardized protocols, variability in study designs, and limited reproducibility of findings.
- Ethical and regulatory issues: concerns over coaching practices, extreme weight-cutting, and athlete wellbeing in high-pressure environments.
- Technological innovations: more research required on the effectiveness of VR and digital training tools in combat sports.
- Public health and policy: exploration of combat sports' role in health initiatives, rehabilitation, and educational programs.

## 5 Conclusion

The evolving discourse on combat sports highlights their potential as a multidimensional tool for wellbeing promotion. While a substantial body of evidence supports their benefits, a critical examination of methodological limitations, scientific hypotheses, and practical applications is essential for further refine our understanding and enhance their effectiveness. Finally, this article makes a significant contribution not only to the field of martial arts but also to public health and sports psychology. Its interdisciplinary approach calls for scientific collaboration and methodological rigor, reinforces the need for evidence-based policies and can serve as a valuable guide for researchers and policymakers looking to integrate combat sports into strategies for promoting health and social inclusion.
